# PD‐1‐Enhanced Treg Cell Senescence in Advanced Maternal Age

**DOI:** 10.1002/advs.202411613

**Published:** 2024-12-23

**Authors:** Guang‐Shun Gong, Yu‐Jing Zhang, Xiao‐Hui Hu, Xin‐Xiu Lin, Ai‐Hua Liao

**Affiliations:** ^1^ Institute of Reproductive Health Center for Reproductive Medicine Tongji Medical College Huazhong University of Science and Technology Wuhan 430030 P. R. China; ^2^ Department of Obstetrics and Gynecology First Clinical College Union Hospital Huazhong University of Science and Technology Wuhan 430022 P. R. China

**Keywords:** adoptive transfer, advance maternal age, cell senescence, maternal‐fetal interface, mice, programmed cell death protein 1, regulatory T cell, senescence‐associated secretory phenotype

## Abstract

Senescence occurs earlier in the immune system than in solid organs as age increases. Regulatory T (Treg) cells are among the first cells to exhibit signs of aging. However, whether advanced‐age pregnancy involves Treg cell aging remains unclear. This study demonstrated that the aging of women is accompanied by aging Treg cells and that PD‐1 regulates Treg cell aging. The transfer of young Treg cells can improve the pregnancy outcomes of reproductive‐aged mice by reducing the level of IFN‐γ, a proinflammatory cytokine secreted by Treg cells in aged mice. Transferring α‐PD‐1 mAb‐treated aged Treg cells increases the level of IL‐10, an anti‐inflammatory cytokine secreted by Treg cells in reproductive‐aged mice. Collectively, these findings suggest a potential therapeutic strategy for preventing adverse pregnancy outcomes in older women.

## Graphical abstract

Gong et al. first discovered that Treg cells in advanced maternal aged pregnancies exhibited cell senescence as well as functional defects. Moreover, PD‐1 may serve as a novel marker of aged Treg cells. Notably, transferring younger Treg cells or aged Treg cells pretreated with anti‐PD‐1 mAbs improves pregnancy outcomes in aged mouse foster mothers. These findings provide a new strategy for preventing adverse pregnancy outcomes in older women.

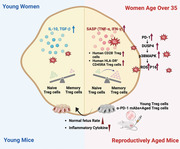

## 1 Introduction

With socioeconomic development and evolving attitudes towards marriage and childbearing, the age at which women conceive has increased significantly worldwide. The International Federation of Obstetricians and Gynecologists defines pregnancy in women over the age of 35 years as advanced maternal age (AMA). AMA is associated with an increased risk of adverse maternal and infant outcomes, including miscarriage, premature delivery, stillbirth, pregnancy complications, and congenital birth defects, which pose serious threats to maternal and infant health. A Danish cohort study that recruited 421201 and 1221546 pregnant women aged 20–30 years found an average early miscarriage rate of approximately 8–10%; the rate increased rapidly after the age of 30 years to 17–25% at the ages of 35–40 years and 33–51% at the ages of 40–45 years. Moreover, it rose as high as 57–75% at the ages of 45 years and older.^[^ ^1^, ^2^ ^]^ Women with AMA have an increased risk of preeclampsia (PE), with those over 40 years of age having a 1.5 to 2.0 times higher risk of PE than younger pregnant women.^[^ ^3^ ^]^ Among pregnant women aged 40 years or older, the risk of miscarriage, PE, gestational diabetes mellitus, small‐for‐gestational‐age neonates, and cesarean section increased by 5.46% compared with those aged 20–34 years.^[^ ^4^ ^]^ Therefore, age is an important risk factor for adverse pregnancy outcomes.

The incidence of adverse pregnancy outcomes increases with increasing age. Many studies have attributed this phenomenon to an exponential increase in chromosomal segregation errors in oocytes with age. However, pregnancy complications and birth defects that occur in older mothers also occur in the absence of embryonic karyotypic abnormalities in humans and mice. Recently, Woods et al.^[^ ^5^ ^]^ reported that abnormal embryonic development in aged female mice was related to severe placentation and decidualization defects and was not only due to defects in the “old” oocytes. Interestingly, the young uterine environment of foster mouse mothers can restore the normal placental and embryonic development of older foster mothers. This finding indicates that an unfavorable maternal uterine environment can hamper normal developmental progression.

During pregnancy, many immune cells are recruited from the periphery to the uterus and are educated by trophoblasts and hormones to form a unique immune microenvironment. This microenvironment at the maternal‐fetal interface protects the fetus from being attacked by the maternal immune system and contributes to placental formation and development. The proportion and function of immune cells at the maternal‐fetal interface changes with maternal age. Levenson et al.^[^ ^6^ ^]^ used a murine model to show that advanced maternal age markedly diminished the number of specific decidual proinflammatory Th1 cell subsets, including CD4^+^ IFN‐γ^+^ and CD8^+^ IFN‐γ^+^ T cells, Th9 cells (CD4^+^IL‐9^+^), and CD4^+^CD25^+^Foxp3^+^ regulatory T (Treg) cells at the maternal‐fetal interface prior to term labor. Our previous study also found that the proportions of effector memory CD4^+^ T cells, terminally differentiated CD4^+^ T cells, and mature NK cells were significantly increased in the peripheral blood in AMA; these cell subsets were closely associated with recurrent pregnancy loss.^[^ ^7^ ^]^

Aging is a natural process that affects all organs, including the immune system. Age‐related changes of the immune system, known as immunosenescence, alter the composition and function of the immune cells. These changes have been implicated in the occurrence and progression of age‐related diseases including cardiovascular disorders, metabolic dysfunction, neuroinflammation, and defective tissue repair and regeneration.^[^ ^8^ ^]^ Treg cells, a subgroup of T cells with immunoregulatory functions, play a crucial role in maintaining the stability of the internal environment and inducing and maintaining immune tolerance during pregnancy. A recent study showed that Treg cell senescence was more severe and occurred earlier than that of effector T cells in the aging process, resulting in the breakdown of the Treg/effector T cell balance and promotion of immune aging.^[^ ^9^ ^]^ However, whether Treg cell senescence occurs in AMA pregnancies and influences pregnancy outcomes remains unclear.

Programmed cell death protein 1 (PD‐1), a key immune checkpoint molecule, plays an important role in immune tolerance by regulating Treg differentiation and function by binding to its ligand. Recently, PD‐1 has been shown to play a promising role in aging. Rivera et al.^[^ ^10^ ^]^ documented hair repigmentation in patients with lung cancer undergoing PD‐1/PD‐L1 inhibitor treatment. These findings suggested a potential link between PD‐1/PD‐L1 inhibitors and aging. Pippin et al.^[^ ^11^ ^]^ reported that PD‐1 was significantly activated in the kidney podocytes of aged mice and humans and that increased PD‐1 expression was positively correlated with declining renal function. Moreover, blocking the PD‐1/PD‐L1 signaling pathway in the brain enhances the excitability, learning, and memory of CA1 neurons in the mouse hippocampus.^[^ ^12^ ^]^ These studies demonstrated a close relationship between PD‐1 and aging. However, whether PD‐1 regulates Treg cell senescence and acts as a novel antiaging agent in AMA pregnancies remains unknown.

Herein, this study aimed to provide a comprehensive overview of the senescence phenotype and function of peripheral and decidual Treg cells in AMA pregnancies. Additionally, we explored the role of PD‐1 in Treg cell senescence in AMA pregnancies and demonstrated that targeting PD‐1 can mitigate Treg cell senescence and improve pregnancy outcomes.

## 2 Results

### 2.1 Cell Senescence Occurred in Human Treg Cells During AMA Pregnancies

**Figure 1:**
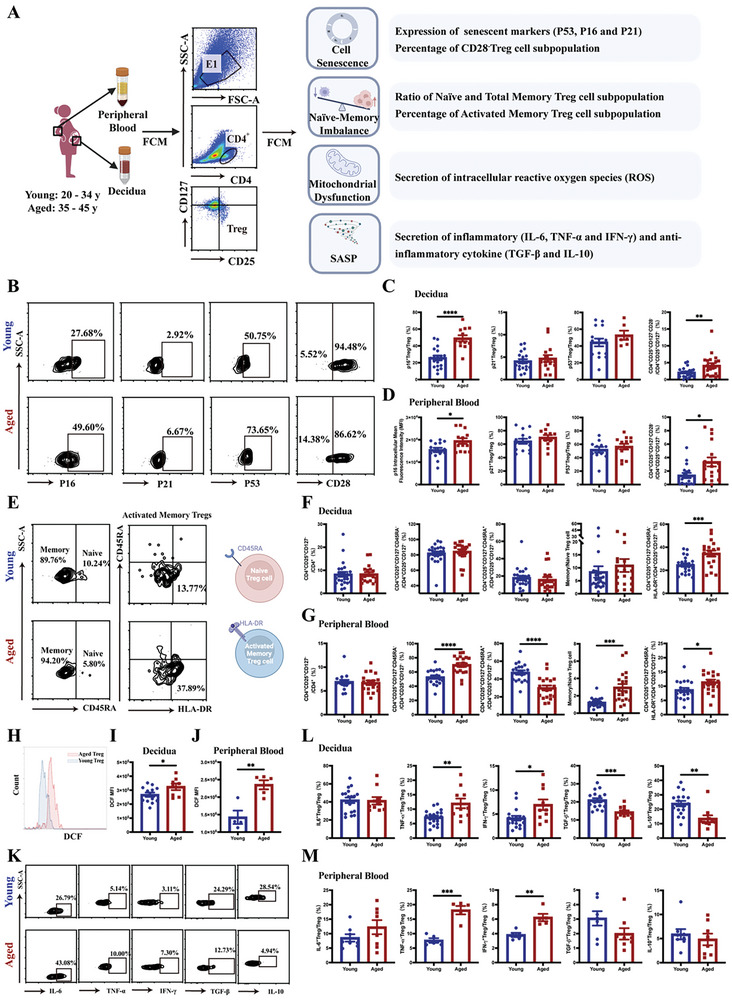
Phenotypic alterations in cell senescence of human Treg cells in advanced maternal age pregnancies. A) Workflow for determining the phenotypic alterations in cell senescence of Treg cells in early pregnancies of young (20–34 y) and old (35–45 y) women. B) Representative flow cytometric images of senescence markers (p16, p21, p53, and CD28) in the decidual Treg cells of the Young and Aged groups. C) Proportions of p16^+^ (Young group: n = 18; Aged group: n = 12), p21^+^(Young group: n = 19; Aged group: n = 15), p53^+^(Young group: n = 15; Aged group: n = 6), and CD28^−^ (Young group: n = 24; Aged group: n = 20) Treg cells within the CD4^+^CD25^+^CD127^−^ Treg cell population in the decidua of the Young and Aged groups. D) MFI of p16^+^ (Young group: n = 14; Aged group: n = 13), proportion of p21^+^(Young group: n = 15; Aged group: n = 11), p53^+^ (Young group: n = 11; Aged group: n = 13), and CD28^−^ (Young group: n = 17; Aged group: n = 17) Treg cells within the CD4^+^CD25^+^CD127^−^ Treg cell population in the peripheral blood of the Young and Aged groups. E) Representative flow cytometric images of the proportions of naïve, memory, and activated memory subpopulations of the decidual Treg cells of the Young and Aged groups. F,G) Proportions of total (CD4^+^CD25^+^CD127^−^), naïve (CD4^+^CD25^+^CD127^−^CD45RA^+^), memory (CD4^+^CD25^+^CD127^−^CD45RA^−^), and activated memory (CD4^+^CD25^+^CD127^−^CD45RA^−^HLA‐DR^+^) Treg cells and the ratio of memory/naïve Treg cells in the decidua (Young group: n = 24; Aged group: n = 19) (F) and peripheral blood (Young group: n = 19; Aged group: n = 20) (G) of the Young and Aged groups. H–J) Representative flow cytometric images (H) and comparison of cellular ROS levels in Treg cells of the decidua (Young group: n = 13; Aged group: n = 8) (I) and peripheral blood (Young group: n = 4; Aged group: n = 5) J) of the Young and Aged groups. K) Representative flow cytometric images of the cytokines (IL‐6, TNF‐α, IFN‐γ, TGF‐β and IL‐10) produced by the decidual Treg cells of the Young and Aged groups. L) Proportions of IL‐6^+^, TNF‐α^+^, IFN‐γ^+^, TGF‐β^+^, and IL‐10^+^ Treg cells within the total Treg cell population in the decidua of the Young (n = 18) and Aged (n = 10) groups. M) Proportions of IL‐6^+^ (Young group: n = 8; Aged group: n = 8), TNF‐α^+^(Young group: n = 5; Aged group: n = 5), IFN‐γ^+^ (Young group: n = 5; Aged group: n = 5), TGF‐β^+^ (Young group: n = 7; Aged group: n = 8), and IL‐10^+^ (Young group: n = 8; Aged group: n = 8) Treg cells within the total Treg cell population in the peripheral blood of the Young and Aged groups. Data are presented as means ± SEM. ****p < 0.0001, ***p < 0.001, **p < 0.01, *p < 0.05.

To evaluate Treg cell senescence in AMA pregnancies, we examined the expression of senescence markers (p16, p21, p53, and CD28) in human Treg cells and compared their functions (producing reactive oxygen species [ROS] and cytokines) during early pregnancy between the Young (20–34 years) and Aged (35–45 years) groups using flow cytometry (FCM) (**Figure** **1**A). The proportions of p16^+^ and CD28^−^ Treg cells in the decidua were significantly higher in the Aged group than in the Young group. However, those of p53^+^ and p21^+^ Treg cells were not significantly different between the two groups (Figure 1B,C). Moreover, the mean fluorescence intensity of p16 in Treg cells and the proportion of CD28^−^ Treg cells in the peripheral blood were significantly increased in the Aged group compared with those in the Young group (Figure 1D).

HLA‐DR is a marker of T cell activation, and activated memory Treg cells increase with age in women, which is detrimental to successful assisted reproductive therapy.^[^ ^13^ ^]^ We previously reported that the proportion of naïve Treg cells (CD4^+^CD25^+^CD127^−^CD45RA^+^ Treg cells) in the peripheral blood decreased with age, whereas the proportion of memory Treg cells (CD4^+^CD25^+^CD127^−^CD45RA^−^ Treg cells) and activated memory Treg cells (CD4^+^CD25^+^CD127^−^HLA‐DR^+^CD45RA^−^ Treg cells) increased with age.^[^ ^13^ ^]^ Therefore, they were selected to further investigate the potential role of the senescent phenotype in young and reproductive‐aged pregnancies. Our results showed that the proportions of total Treg cells in the peripheral blood and decidua were not significantly different between the Young and Aged groups (Figure 1F,G). Compared with the Young group, the proportions of activated memory Treg cells in both the peripheral blood and decidua of the Aged group were significantly increased (Figure 1E–G). The proportion of total memory Treg cells and the ratio of memory/naïve Treg cells were also significantly increased, while that of naïve Treg cells was significantly decreased in the peripheral blood of the Aged group (Figure 1G).

Oxidative stress is an important indicator of immune system senescence. In this study, the intracellular ROS levels of Treg cells in the peripheral blood and decidua were detected using FCM (Figure 1H). Our results showed that the intracellular ROS levels in Treg cells were significantly higher in the peripheral blood and decidua of the Aged group than in the Young group (Figure 1I,J).

Senescence‐associated secretory phenotype (SASP) is a key characteristic that distinguishes senescent cells from apoptotic cells. To further elucidate the differences in the SASP of Treg cells in young and reproductive‐aged pregnant women, we utilized FCM to detect the levels of senescence‐associated proinflammatory cytokines (IL‐6, TNF‐α, and IFN‐γ) and anti‐inflammatory cytokines (IL ‐10 and TGF‐β) (Figure 1K). The proportions of TNF‐α^+^ and IFN‐γ^+^ Treg cells were significantly increased in both the decidua and peripheral blood of the Aged group compared with those of the Young group. However, the proportions of IL‐6^+^ Treg cells in the decidua and peripheral blood were not significantly different between the two groups (Figure 1L,M). Conversely, the proportions of IL‐10^+^ and TGF‐β^+^ Treg cells in the decidua but not in the peripheral blood were significantly decreased in the Aged group when compared with those in the Young group (Figure 1L). These findings indicate that Treg cells exhibit an aging phenotype during AMA pregnancies.

### 2.2 Cell Senescence Occurred in Treg Cells During Advanced Maternal Age Pregnancies in Mice

**Figure 2:**
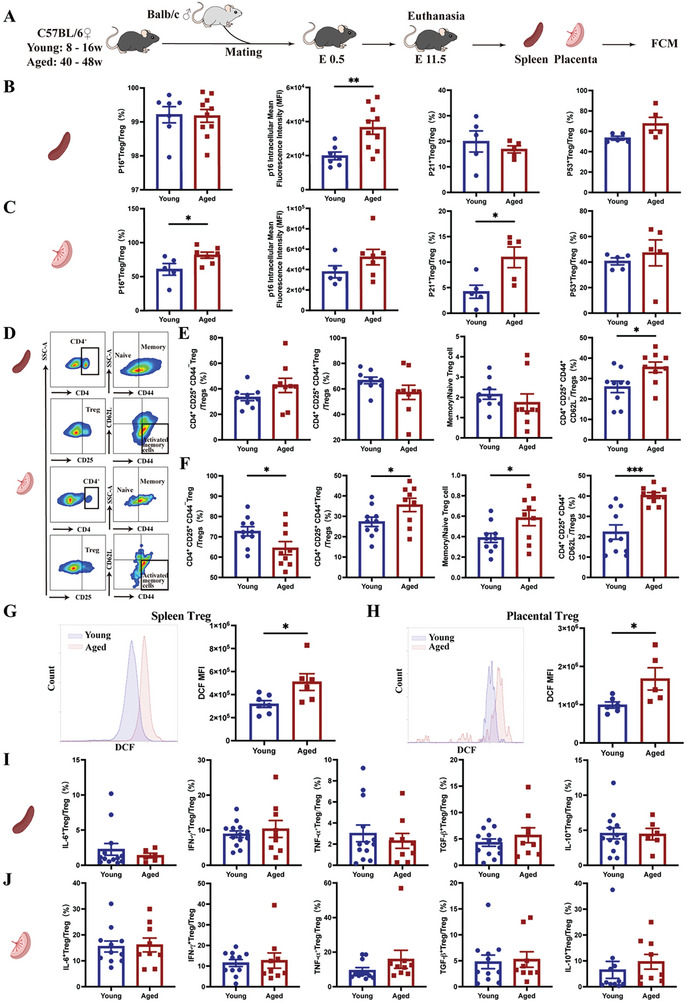
Cell senescence in Treg cells during aged murine pregnancies. A) Mating strategy to establish a pregnancy model in young and reproductive‐aged mice. B) Proportions of p53^+^ (Young group: n = 5; Aged group: n = 5), p21^+^ (Young group: n = 5; Aged group: n = 5), and p16^+^ (Young group: n = 7; Aged group: n = 10) Treg cells within the total population of CD4^+^CD25^+^ Treg cells in splenic tissue from the Young and Aged groups. C) Proportions of p53^+^ (Young group: n = 5; Aged group: n = 5), p21^+^ (Young group: n = 5; Aged group: n = 5), and p16^+^ (Young group: n = 5; Aged group: n = 7) Treg cells within the total population of CD4^+^CD25^+^ Treg cells in placental tissue from the Young and Aged groups. D) FCM gating strategy and representative flow cytometric images of naïve, memory, and activated memory Treg cell subpopulations in the spleen and placenta of the Young and Aged groups. E,F) Proportions of naïve (CD4^+^CD25^+^CD44^−^), memory (CD4^+^CD25^+^CD44^+^), activated memory (CD4^+^CD25^+^CD62L^−^CD44^+^) Treg cells and ratio of memory/naïve Treg cells in the splenic (Young group: n = 9; Aged group: n = 9) (E) and placental (Young group: n = 10; Aged group: n = 9) (F) tissues. (G–H) Representative flow cytometric images and cellular ROS levels in Treg cells in splenic (Young group: n = 7; Aged group: n = 6) G) and placental (Young group, n = 6; Aged group, n = 5) H) tissues of the Young and Aged groups. I) Quantitative analysis of the production of IL‐6^+^ (Young group: n = 13; Aged group: n = 6), TNF‐α^+^ (Young group: n = 13; Aged group: n = 9), IFN‐γ^+^ (Young group: n = 13; Aged group: n = 9), IL‐10^+^ (Young group: n = 13; Aged group: n = 6), and TGF‐β^+^ (Young group: n = 13; Aged group: n = 9) by splenic Treg cells of the Young and Aged groups. J) Quantitative analysis of the production of IL‐6^+^, TNF‐α^+^, IFN‐γ^+^, IL‐10^+^, and TGF‐β^+^ by placental Treg cells of the Young (n = 11) and Aged (n = 9) groups. Data are presented as means ± SEM. ***p < 0.001, **p < 0.01, *p < 0.05. Treg cells: CD4^+^CD25^+^.

To further confirm the cell senescence occurring in Treg cells in AMA pregnancies, we established young and aged pregnant mouse models (Young group: C57BL/6 female mice at 8–16 weeks of age mated with Balb/c male mice at 8–16 weeks of age; Aged group: C57BL/6 female mice at 40–48 weeks of age mated with Balb/c male mice at 8–16 weeks of age). At E11.5, pregnant mice were euthanized, and the spleen and placenta were collected to detect the expression of senescence markers (p16, p21, and p53) in Treg cells using FCM (**Figure** **2**A).

In the spleen, apart from the increased p16^+^ MFI in the Aged group, no significant differences were observed in the proportions of p16^+^, p21^+^, and p53^+^ Treg cells between the two groups (Figure 2B). Compared with the Young group, the proportions of p16^+^ and p21^+^ Treg cells were significantly increased in the placentas of the Aged group, while no significant differences in the p16^+^ MFI and proportion of p53^+^ Treg cells were observed in the placentas of both groups (Figure 2C).

As in the aforementioned human study, we detected the proportions of naïve (CD4^+^CD25^+^CD44^−^), memory (CD4^+^CD25^+^CD44^+^) and activated memory (CD4^+^CD25^+^CD62L^−^CD44^+^) Treg cell subsets in both mouse spleens and placentas using FCM (Figure 2D). The proportion of activated memory Treg cells in the spleens of the Aged group was significantly higher than that in the spleens of the Young group (Figure 2E). However, the proportions of naïve and memory Treg cells and the ratio of memory/naïve Treg cells in the spleen were not significantly different between the two groups. In addition, the proportion of naïve Treg cells in the placenta was significantly lower in the Aged group than in the Young group, whereas the proportion of total memory Treg cells was higher (Figure 2F). Consistent with the human studies, the ratio of memory/naïve Treg cells and the proportion of activated memory Treg cells were significantly increased in the placentas of the Aged group compared to those in the placentas of the Young group (Figure 2F).

The intracellular ROS levels in Treg cells of the spleen and placenta were also higher in the Aged group than in the Young group (Figure 2G,H). However, no significant difference was observed in the levels of senescence‐associated proinflammatory (IL‐6, TNF‐α, and IFN‐γ) and anti‐inflammatory (IL‐10 and TGF‐β) cytokines secreted by Treg cells in the spleen and placenta between the two groups (Figure 2I,J). These findings indicate that, similar to the human results, Treg cells in the Aged group of mice exhibited aging.

### 2.3 PD‐1 Regulated Treg Cell Senescence

**Figure 3:**
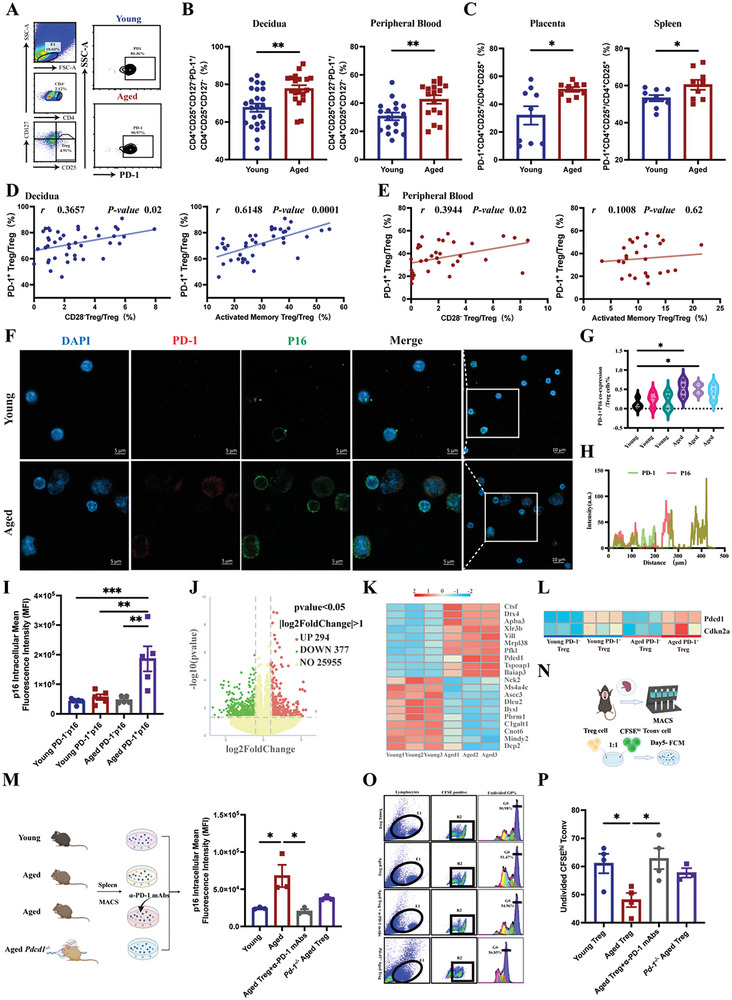
PD‐1 regulated Treg cell senescence. A) FCM gating strategy and representative flow graph of PD‐1^+^ Treg cells in decidual tissues. B) Proportions of PD‐1^+^ Treg cells in the total CD4^+^CD25^+^CD127^−^ Treg cell population in the decidua and peripheral blood of the Young (n = 24) and Aged (n = 19) groups. C) Proportions of PD‐1^+^Treg cells within the total CD4^+^CD25^+^ Treg cell population in the splenic and placental tissues of the Young (n = 9) and Aged (n = 9) groups. D,E) Pearson's correlation analysis of the proportion of PD‐1^+^ and senescent Treg cells (CD4^+^CD25^+^CD127^−^CD28^−^ and CD4^+^CD25^+^CD127^−^CD45RA^−^HLA‐DR^+^, respectively) in decidual tissues (D) and peripheral blood (E). F) Representative plots of co‐localized fluorescence of PD‐1 and p16 in splenic Treg cells of the Aged and Young group of mice. G) Plots of statistical analysis of PD‐1^+^p16^+^ Treg cell counts of two groups of mice. H) Statistical plots of quantitative analysis of co‐localized fluorescence of PD‐1 and p16 in Treg cells from two groups of mice. I) Average fluorescence intensity of p16 expression in PD‐1^+^ Treg cells from two groups of mice by FCM. J) Volcano plot of differentially expressed genes in mouse splenic Treg cells (each n = 3) (pvalue < 0.05, ǀfold changeǀ> 1). K) Cluster heatmap of differentially expressed genes in mouse splenic Treg cells, red (high levels), blue (low levels). L) Cluster heatmap of *Cdkn2a* and *Pdcd1* expressed in PD‐1^+^ and PD‐1^−^ Treg cells, red (high levels), blue (low levels). M) Expression of senescence marker p16 on Treg cells of mice in four groups. To determine whether the aging of Treg cells depends on PD‐1 regulation, primary Treg cells were isolated from the spleens of mice and divided into four groups: (1) Young group: primary Treg cells from the spleens of young mice; (2) Aged group: primary Treg cells from the spleens of aged mice; (3) Aged Treg^+^ α‐PD‐1 mAbs group: primary Treg cells from the spleens of aged mice pre‐treated with 10 µg mL^−1^ α‐PD‐1 mAbs for 24 h; (4) Pdcd1^−/−^ Aged group: primary Treg cells from the spleens of *Pdcd1*‐knockout aged mice. N) Workflow of Treg cell suppression assay. O) Representative plots of Tconv cell proliferation in each group using NovaExpress software. P) Comparison of nonproliferating Tconv cells to the total CFDA^+^ Tconv cell population in each group as the same as Figure 3M described. Data are presented as means ± SEM. ***p < 0.001, **p < 0.01, *p < 0.05. Pearson correlation analysis was used, and r represents the correlation coefficient.

PD‐1, a key immune checkpoint molecule, is associated with aging. PD‐1 expression in mouse T cells increases with aging and is associated with age‐related T cell dysfunction.^[^ ^14^ ^]^ To determine whether PD‐1 is involved in Treg cell senescence in AMA pregnancies, we first detected and compared the proportion of human PD‐1^+^ Treg cells in the decidua and peripheral blood of the two groups using FCM (**Figure** **3**A). The proportions of human PD‐1^+^ Treg cells in the decidua and peripheral blood of the Aged group were significantly higher than those in the Young group (Figure 3B). Similar results were observed in mice (Figure 3C).

Since CD28^−^ and activated memory (HLA‐DR^+^CD45RA^−^) Treg cell subsets exhibited senescent phenotypes, we further analyzed the correlation between these two Treg cell subsets and human PD‐1^+^ Treg cells using Pearson correlation analysis. Our results demonstrated a positive correlation between the proportion of PD‐1^+^ Treg cells and that of CD28^−^ Treg cells (*r* = 0.3657, *p* < 0.05) and HLA‐DR^+^CD45RA^−^ Treg cells (*r* = 0.6148, *p* < 0.0001) in human decidua (Figure 3D). In the peripheral blood, the proportion of PD‐1^+^ Treg cells was also positively correlated with that of CD28^−^ Treg cells (*r* = 0.3944, *p* < 0.05) but not with that of HLA‐DR^+^CD45RA^−^ Treg cells (Figure 3E).

To investigate the role of PD‐1 in senescent Treg cells, we selected p16 as a cell senescence marker and explored the relationship between PD‐1 and p16 expression in the Young and Aged groups of mice in vitro. Mouse splenic Treg cells were purified by magnetic‐activated cell sorting (MACS), and the expression of PD‐1 and p16 in these cells was assessed by immunofluorescence (IF) and FCM, respectively. IF results showed that the co‐localization of p16 with PD‐1 in mouse splenic Treg cells was significantly increased in the Aged group compared to that in the Young group (Figure 3F–H). The FCM results showed that the MFI of p16 expression in PD‐1^+^ Treg cells was significantly higher in the Aged group than that in the Young group (Figure 3I).

To further investigate the correlation between the expression of aging‐related genes and PD‐1 expression in aged Treg cells, we performed RNA sequencing on mouse spleen Treg cells from the Aged and Young groups. Transcriptome sequencing identified 671 significantly differentially expressed genes between the Young and Aged groups, with 294 upregulated and 377 downregulated genes (Figure 3J). The cluster heatmap of the differentially expressed genes in Treg cells showed that genes such as *Ctsf*, *Dtx4, Apba3*, and *Pdcd1* were significantly enriched in the Aged group, whereas other genes, including *NCk2, Pbrm1*, and *Dcp2*, were significantly enriched in the Young group (Figure 3K). Furthermore, the senescent gene *Cdkn2a* (encoding the p16 protein) was enriched in the Aged PD‐1^+^ Treg group compared to that in the Aged PD‐1^−^ Treg group (Figure 3L). FCM assay showed that the mean fluorescence intensity of p16 was significantly decreased in the Aged Treg group pre‐treated with α‐PD‐1 mAbs compared to that in the Aged Treg group (Figure 3M), demonstrating that α‐PD‐1 mAbs could reduce Treg cell senescence.

Additionally, we examined changes in the immunosuppressive function of Treg cells inhibiting Tconv cell proliferation under four treatment conditions as Figure 3M described to evaluate the effect of PD‐1 on Treg cell function. After co‐culturing mouse splenic Treg cells in the four groups with splenic aged Tconv cells for 5 days, the proliferation capacity of aged Tconv cells was assessed using FCM (Figure 3N). The proportion of undivided Tconv cells was significantly lower in the Aged Treg group than in the Young Treg group. However, in the Aged Treg group pretreated with α‐PD‐1 mAbs, the proportion of undivided Tconv cells was significantly increased compared to that in the Aged Treg group. However, the proportion of undivided Tconv cells in the *Pdcd1* ^−/−^ Aged group was increased compared to that in the Aged Treg group, although the difference was not statistically significant (Figure 3O,P).

### 2.4 Characterization of Young and Aged PD‐1^+^ and PD‐1^−^ Treg Cells

**Figure 4:**
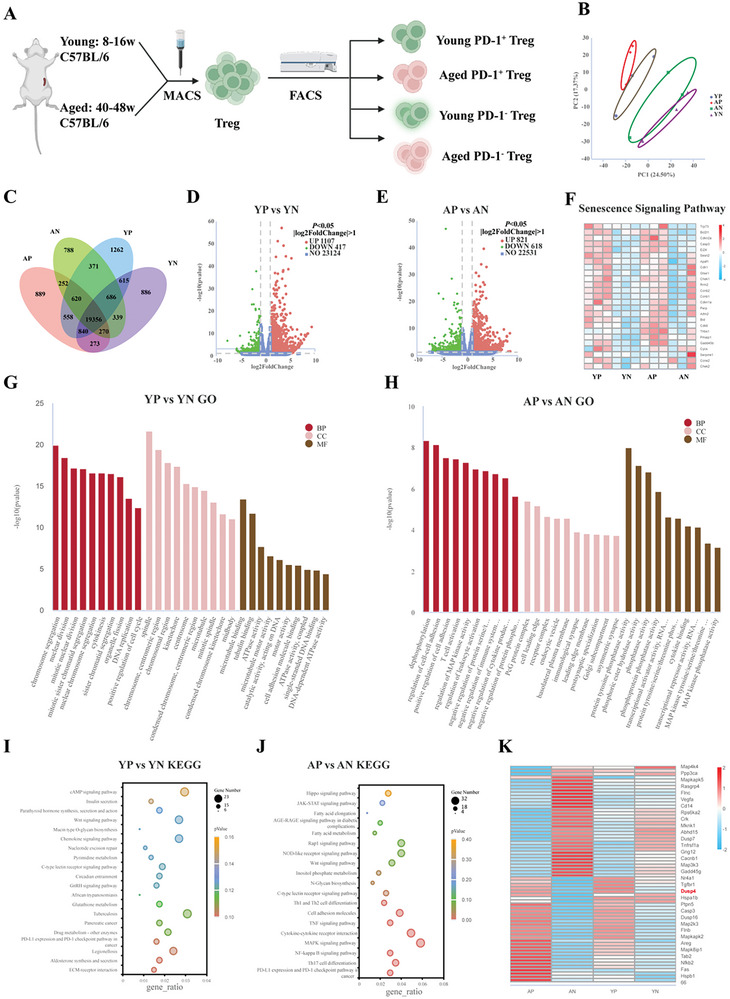
Characterization of young and aged PD‐1^+^ and PD‐1^−^ Treg cells. A) Isolation strategy for PD‐1^+^ and PD‐1^−^ Treg cells in the spleen of mice. B) Principal component analysis of the genes in each group. C) Analysis of the Venn diagram of the gene co‐expression in each group of samples. D,E) Volcano plot of differentially expressed genes in PD‐1^+^ and PD‐1^−^ Treg cells in the Young (D) and Aged (E) groups (pvalue < 0.05, ǀfold changeǀ> 1). F) Cluster heatmap of differentially expressed senescence pathway‐related genes, red (high levels), blue (low levels). G,H) GO analysis and histogram of differentially enriched genes in PD‐1^+^ and PD‐1^−^ Treg cells in the Young (G) and Aged (H) groups. I,J) KEGG signaling pathway analysis of differentially expressed genes in PD‐1^+^ and PD‐1^−^ Treg cells of the Young (I) and Aged (J) groups. Dot size reflects the ratio of identified gene numbers that change between two groups. Dot color represents the pvalue, yellow (high values), red (low values) K) Cluster heatmap of differentially expressed MAPK pathway‐related genes, red (high levels), blue (low levels). YP: PD‐1^+^ Treg cells in the Young group; YN: PD‐1^−^ Treg cells in the Young group; AP: PD‐1^+^ Treg cells in the Aged group; AN: PD‐1^−^ Treg cells in the Aged group; MF: molecular function, CC: cellular component, BP: biological process.

To explore the transcriptomic signatures of PD‐1^+^ and PD‐1^−^ Treg cells, we sorted PD‐1^+^ and PD‐1^−^ Treg cells from the spleens of mice in the Young and Aged groups using MACS and FACS and performed transcriptome sequencing analysis (**Figure** **4**A). Principal component analysis of sequencing showed that the four groups of samples were dispersed among the groups, and the samples within the groups were aggregated (Figure 4B). The PD‐1^+^ Treg cells of the Aged group (AP group) expressed 889 unique genes, whereas the PD‐1^−^ Treg cells in the Aged group (AN group) expressed 788. The PD‐1^+^ Treg cells in the Young group (YP group) expressed 1262 unique genes, and the PD‐1^−^ Treg cells in the Young group (YN group) expressed 886. The number of genes expressed in all four groups was 19356 (Figure 4C). Transcriptome sequencing identified 1524 significantly differentially expressed genes between the YP and YN groups, with 1107 upregulated and 417 downregulated genes (Figure 4D). Moreover, 1439 genes were significantly differentially expressed between the AP and AN groups, with 821 upregulated and 618 downregulated genes (Figure 4E).

The gene expression cluster heatmap for genes related to cellular senescence pathways showed that the expression of senescence‐related genes was upregulated in the YP and AP groups compared to that in the YN and AN groups (Figure 4F). Bioinformatics enrichment analysis of differential genes in the YP and YN groups showed 335 statistically different gene ontology (GO) signaling pathways, of which DNA replication activity (GO:0 006260), chromosome‐associated cellular components (GO:0000775), and DNA catalytic active molecular processes (GO:014 0097) were significantly upregulated (Figure 4G). Bioinformatic enrichment analysis of differentially expressed genes in the AP and AN groups showed 313 statistically different GO signaling pathways, including mitogen‐activated protein kinase (MAPK) activity (GO:00 43405) and MAPK tyrosine/serine/threonine kinase activity (GO:0 008138), that were significantly upregulated (Figure 4H).

Kyoto Encyclopedia of Genes and Genomes (KEGG) enrichment analysis of differential genes in the YP and YN groups showed 24 significantly different signaling pathways, of which cytokine‐receptor interactions, the p53 signaling pathway, and the cell cycle pathway were significantly enriched in the YP group (Figure 4I). KEGG enrichment analysis of differential genes in the AP and AN groups showed 30 significantly different signaling pathways, of which the MAPK pathway and pathways related to inflammation (tumor necrosis factor α signaling pathway), and cell growth inhibition (Hippo signaling pathway) were significantly enriched in the AP group (Figure 4J). Sun et al.^[^ ^15^ ^]^ reported that dual‐specificity phosphatase (DUSP) regulated the functional properties of T cells. Among the different DUSPs, DUSP4 negatively engages in maintaining the homeostasis of Treg subsets. Moreover, increased DUSP4 expression has been observed in aged CD4^+^ T cells.^[^ ^16^ ^]^ Cluster analysis of differentially expressed genes between PD‐1^+^ and PD‐1^−^ Treg cells in the spleen tissues of young and aged mice revealed that *DUSP4* was enriched in the PD‐1^+^ Treg cell population, suggesting that DUSP4 may regulate MAPK signaling by dephosphorylating phosphorylated threonine, serine, and tyrosine (Figure 4K).

### 2.5 Fetal Defects and Poor Placentation in Aged Mouse Models

**Figure 5:**
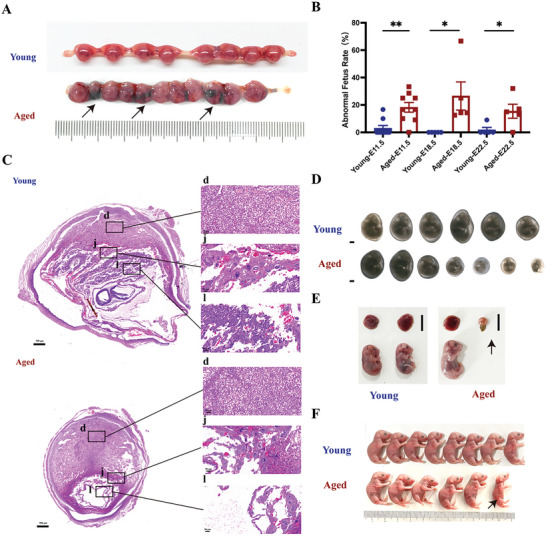
Fetal defects and poor placentation in mouse models of AMA pregnancy. A) Representative images showing comparative litter size and uterine colors between the Young and Aged groups at E11.5. B) Abnormal fetus rates at E11.5 (Young group: n = 9; Aged group: n = 9), E18.5 (Young group: n = 5; Aged group: n = 5), and after birth (E22.5) (Young group: n = 5; Aged group: n = 5), respectively. C) Representative H&E images of placentas at E11.5 (scale bar, 500 µm). D–F) Representative images of fetuses at E11.5 (scale bar, 1 mm) (D), E18.5 (scale bar, 1 cm) (E), and after birth (scale bar, 1 cm) (F). Data are presented as means ± SEM. **p < 0.01, *p < 0.05. D: decidual layer; J: junctional layer; L: labyrinthine layer.

C57BL/6 (B6) females at approximately 48 weeks of age are nearing the end of their reproductive lifespan but can still become pregnant. Fetal defects, including hemorrhage and resorption, were more obvious in the Aged group than in the Young group at E11.5 (**Figure** **5**A). At E11.5, in the Aged group, 18.26% of the fetus had defective development, whereas in the Young group, 2.96% had (Figure 5B). At E18.5, the fetal loss rates were higher in the Aged group compared to the Young group (Figure 5B). On the day after birth, abnormally small fetus with lower body weights were higher in the Aged group than in the Young group (Figure 5B). Hematoxylin and eosin (HE) staining showed that at E11.5, the Young group had a clear demarcations between different placental layers, with well‐developed vasculature in the labyrinthine layer, which facilitated the maternal‐fetal exchange of nutrients and waste (Figure 5C). In contrast, the placental volume at the absorption site in the Aged group was reduced, and demarcation of the placental layer was not obvious (Figure 5C). Moreover, sparse labyrinthine structures and widespread maternal erythrocytes were observed in the Aged group (Figure 5C), demonstrating that ruptured maternal blood vessels, hemorrhage, and poor placental development might lead to insufficient nutrients and oxygen for fetal development. Moreover, the fetus were dissected, fixed in 4% paraformaldehyde and photographed under a dissecting microscope at E11.5. Observations of the gross morphological appearance revealed increased developmental variability with severe developmental delay, malformation, and edema in the litters of the Aged group compared with those of the Young group at E11.5 (Figure 5D). Fetal abnormalities, including small fetal size and resorption, were observed in the Aged group at E18.5 and after birth, with resorption noticeable at E18.5 (Figure 5E) and smaller fetus (Figure 5F) apparent postnatally.

### 2.6 Adoptive Transfer of Treg Cells in the AMA Mouse Model

**Figure 6:**
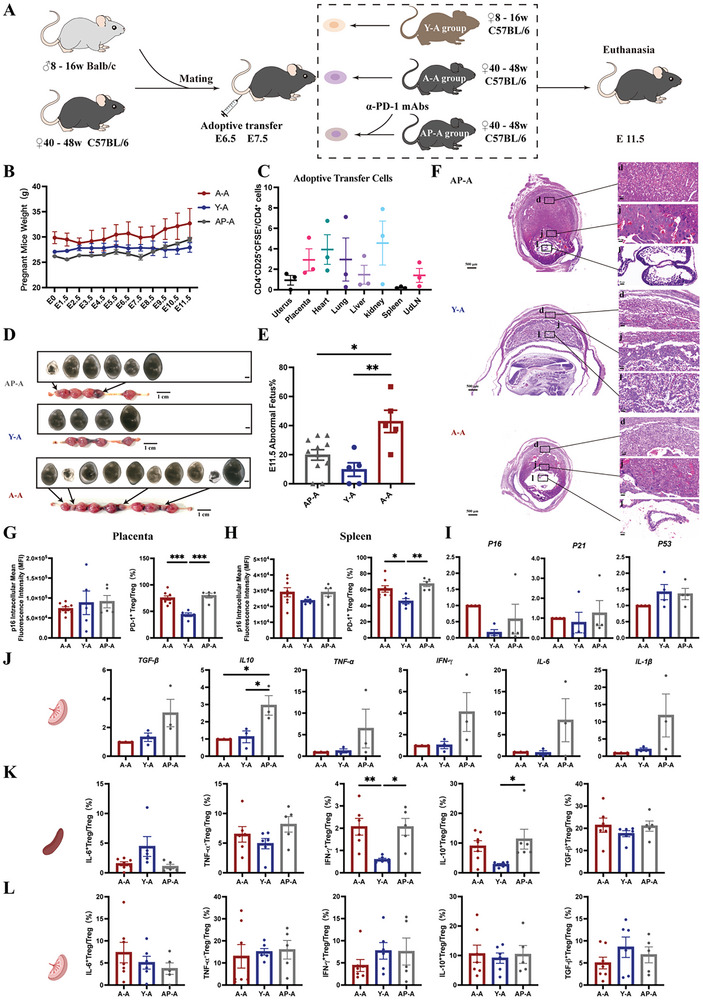
Adoptive transfer of Treg cells in a mouse model of AMA pregnancy. A) Mating strategy to establish a model of adoptive transfer in the Young and Aged groups. B) Body weight progression of female C57BL6/J mice evaluated every day (n = 3). C) Proportions of labeled Treg cells (CD4^+^CD25^+^CFSE^+^) within the total CD4^+^ cell population of different organs (n = 3 per group). D) Representative images of the uterus and macroscopic view of the fetus at E11.5. E) Proportions of abnormal fetus rates at E11.5 in AP‐A group (n = 11), Y‐A group (n = 5) and A‐A group (n = 5). F) Representative images of H&E staining of the placenta at E11.5. (G) Comparison of Treg cells expressing p16 and PD‐1 in placental G) and splenic H) tissues using FCM in A‐A group (n = 8), Y‐A group (n = 5) and AP‐A group (n = 5). I) mRNA expression levels of *p16, p21*, and *p53* in placental tissues (n = 4 per group). J) Comparison of mRNA expression of *TGF‐β, IL‐10, TNF‐α, IFN‐γ*, *IL‐6*, and *IL‐1β* in placental tissues (n = 3 per group). K) Proportions of IL‐6^+^(A‐A group, n = 7; Y‐A group, n = 5; AP‐A group, n = 5), IL‐10^+^(A‐A group, n = 7; Y‐A group, n = 6; AP‐A group, n = 5), TNF‐α^+^, IFN‐γ^+^ and TGF‐β^+^(A‐A, n = 6; Y‐A, n = 6; AP‐A, n = 5) Treg cells within the total Treg cell population in splenic tissues. L) Proportions of IL‐6^+^, TNF‐α^+^, IFN‐γ^+^, IL‐10^+^, and TGF‐β^+^ Treg cells within the total Treg cell population in placental tissues in A‐A group (n = 7), Y‐A group (n = 6) and AP‐A group (n = 5). Data are presented as means ± SEM. ***p < 0.001, **p < 0.01, *p < 0.05.

Blockade of PD‐1 signaling enhances the inhibitory function and decreases the expression of senescence markers in Treg cells in aged mice. To further clarify the link between PD‐1 and advanced pregnancy, we examined the effect of the adoptive transfer of Treg cells on the pregnancy outcomes in aged mice and its possible mechanism (**Figure** **6**A–C). Compared with the A‐A group (Treg cells transferred from an aged mouse to an aged mouse), the abnormal fetus rate was significantly decreased in both the Y‐A (Treg cells transferred from a young mouse to an aged mouse) and AP‐A (α‐PD‐1‐treated Treg cells from an aged mouse transferred to an aged mouse) groups (Figure 6D,E). HE staining of mouse placentas showed improved development in the Y‐A group compared to that in the A‐A group. In the Y‐A group, the meconium, junctional, and labyrinthine layers had an orderly arrangement, with well‐developed blood vessels (Figure 6F). We also detected the expression of the senescence marker p16 in Treg cells in each group. The mean fluorescence intensity of p16 in Treg cells from the spleen and placenta was detected using FCM, and no statistically significant differences were found among the groups (Figure 6G,H). However, the proportion of PD‐1^+^ Treg cells among the total Treg cell population was lowest in the spleen and placenta of the Y‐A group (Figure 6G,H).

To clarify the possible mechanism by which adoptive transfer affects adverse pregnancy outcomes in aged mice, we measured the mRNA expression levels of the senescence‐related genes *p16*, *p21*, and *p53* in Treg cells from placental tissues of recipient‐aged pregnant mice at E11.5. The differences in mRNA levels were not statistically significant (Figure 6I). Moreover, we analyzed the levels of inflammatory cytokines (IL‐6, TNF‐α, IFN‐γ, and IL‐1β) and inflammation‐suppressing cytokines (IL‐10 and TGF‐β) secreted by Treg cells in placental tissues. The results showed an increased mRNA level of the anti‐inflammatory factor IL‐10 in the placenta of the AP‐A group compared with that in the A‐A group, which may explain the improvement in pregnancy outcomes in the Aged group (Figure 6J). The proportion of IFN‐γ^+^ Treg cells in the spleen was significantly lower in the Y‐A group than in the A‐A group. The proportion of IL‐10^+^ Treg cells in the spleen was significantly higher in the AP‐A group than in the Y‐A group (Figure 6K,L).

## 3 Discussion

**Figure 7:**
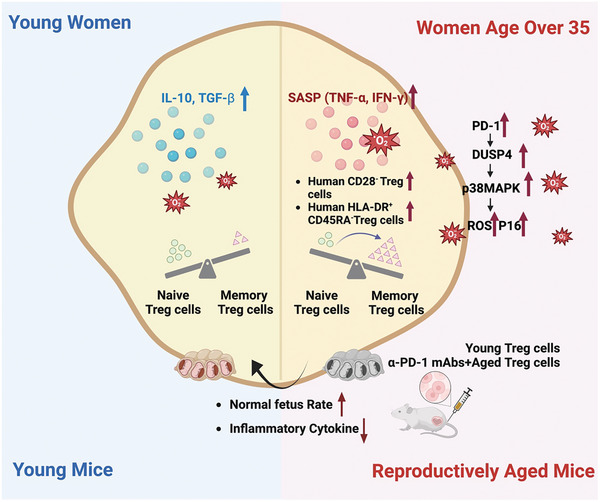
Schematic of Treg cell aging and regulatory role of PD‐1 in advanced‐age pregnancies. Treg cells exhibit senescent phenotypes in advanced‐age pregnancies in humans and mice, which are characterized by increased levels of P16 and ROS, SASP, increased expression of PD‐1, and a naïve/memory Treg cell balance that is biased to memory Treg cells. The proportion of PD‐1^+^ Treg cells are positively correlated with Treg cells with an aging phenotype (CD28^−^ and HLA‐DR^+^CD45RA‐Treg cells). The ability of aged Treg cells to suppress effector T cells is also decreased. PD‐1 positively regulates *DUSP4* in aged Treg cells, which activates the p38 MAPK signaling pathway. The active transfer of young Treg cells or aged Treg cells pretreated with α‐PD‐1 mAbs effectively reduces the incidence of abnormal fetuses and improves the placental development in a reproductive‐aged mouse model. ROS: intracellular reactive oxygen species; SASP: senescence‐associated secretory phenotype; DUSP4: dual specificity phosphatase 4; MAPK: mitogen‐activated protein kinase. Figure 7 was generated using Biorender.com.

Age is an independent influencing factor of advanced‐age pregnancy outcomes. The risk of advanced‐age pregnancy complications increases with age, including premature delivery, fetal growth restriction, PE, and pregnancy diabetes.^[^ ^17^, ^18^ ^]^ Immune aging is a popular research topic that is involved in the occurrence and development of various age‐related diseases.^[^ ^19^ ^]^ However, research on immune aging at the maternal‐fetal interface is scarce. This study is the first to demonstrate that Treg cells in AMA pregnancies exhibit cell senescence as well as functional defects, which could contribute to adverse pregnancy outcomes. Moreover, PD‐1 may serve as a novel marker of aged Treg cells. Blocking PD‐1 signaling in aged Treg cells can restore their aging status and function. Notably, transferring younger Treg cells or aged Treg cells pretreated with anti‐PD‐1 mAbs improves pregnancy outcomes in aged mouse foster mothers (**Figure** **7**).

Cell‐cycle arrest is one of the main characteristics of cell senescence. The cyclin‐dependent kinase inhibitors p21 and p16 are commonly used as markers to evaluate the cell cycle arrest caused by DNA damage.^[^ ^20^, ^21^ ^]^ Our study found that the average fluorescence intensity of p16 in Treg cells of both women and mice with an advanced maternal age was markedly increased. This is consistent with a study by Guo et al.,^[^ ^9^ ^]^ which showed that the Treg cells of older mice had increased p16 and p21 expression. In addition, we found an abnormal increase in the proportion of CD28^−^ Treg cells (an aging phenotype) in advanced‐age pregnancies. CD28^−^ T cells are mainly found at chronic inflammatory sites and are widely distributed in the peripheral circulation during aging. These are considered typical phenotypic changes associated with the aging of T cells.^[^ ^22^ ^]^

Compared with memory Treg (CD45RA^−^ Treg) cells, naïve Treg cells (CD45RA^+^ Treg) have long telomeres, increased T‐cell receptor (TCR) diversity, and low proliferative potential.^[^ ^23^ ^]^ Our study also revealed an abnormally high proportion of HLA‐DR^+^CD45RA^−^ Treg cells in the decidua and peripheral blood of humans with advanced‐age pregnancies, which is consistent with our previous and other studies.^[^ ^24^, ^25^ ^]^ HLA‐DR^+^CD45RA^−^ Treg cells are a subpopulation of Treg cells that can rapidly expand at an inflammatory state and are characterized by an impaired suppressive capacity and early senescence.^[^ ^26^ ^]^ Recently, our group analyzed and compared the differences in peripheral HLA‐DR^+^CD45RA^−^ Treg cells in women with ages of 20–29, 30–34, and 35–39 years. The results showed that the proportion of HLA‐DR^+^CD45RA^−^ Treg cells significantly increased with age and has a significant negative correlation with indicators of ovarian reserve, including anti‐Müllerian hormone levels and antral follicle counts.^[^ ^13^ ^]^ Another study by Schlossberger et al.^[^ ^24^ ^]^ sorted HLA‐DR^+^CD45RA^−^ Treg cells in the peripheral blood of pregnant and nonpregnant women to assess their suppressive function. Their results showed that the suppressive capacity of HLA‐DR^+^CD45RA^−^ Treg cells was significantly reduced in pregnant women compared to that in nonpregnant women, which demonstrates that the increase in HLA‐DR^+^CD45RA^−^ Treg cells in the decidua of advanced‐age women may be unfavorable for the induction of maternal‐fetal immune tolerance in early pregnancy. This is consistent with our results that demonstrated that the suppressive ability of Treg cells on effective T cells decreased in AMA pregnancies. Guo et al.^[^ ^9^ ^]^ also found that Treg cells from aged (>18 months) mice were less efficient than young Treg cells in suppressing Tconv cell functions in a model of inflammatory bowel disease and preventing Tconv cell aging in a model of irradiation‐induced aging. In mice, CD62L^−^CD44^+^ cells are used to identify activated memory Treg cells, and our results were similar to those of the human study.

Intracellular ROS levels are strictly regulated by redox reactions, and disruption of the ROS balance may lead to excessive ROS, which can cause cell senescence and aging. Here, we discovered that ROS levels were increased in Treg cells in both the peripheral blood and decidua of older pregnant women compared to those of young pregnant women. Similar results were observed in the spleens and placentas of aged pregnant mice. Mitochondria are signal hubs that produce, transmit, and respond to ROS, and their functions are critical for cellular energy and metabolism. Therefore, at an advanced age, the mitochondrial function of Treg cells decreases, and cellular oxidative stress increases. Guo et al.^[^ ^9^ ^]^ compared the ROS levels in Treg cells of young (3 months old) and aged (18 months old) mice and found that the ROS level in Treg cells of aged mice increased, which is similar to our mouse study results. However, to the best of our knowledge, no studies have reported that human Treg cells in older women with early pregnancies produce higher ROS levels than young Treg cells.

Senescent cells contribute to inflammaging via their SASP. We observed increased levels of SASP produced by Treg cells in the peripheral blood and decidua of older pregnant women, further suggesting that pregnancy in older women is accompanied by Treg cell aging rather than exhaustion. Although senescent and exhausted T cells have overlapping features and similar proliferation defects and expression of cell cycle arrest markers, they differ in their molecular signaling and secretory phenotypes. Senescent T cells secrete large amounts of SASP, including proinflammatory cytokines (IL‐6, TNF‐α, and IFN‐γ), chemokines (CCL2 and IL‐8), growth factors (fibroblast growth factor), and matrix metalloproteinases (metalloproteinases 1 and 3).^[^ ^27^ ^]^ In this study, we found that aged Treg cells in the peripheral blood produced higher levels of proinflammatory cytokines, such as TNF‐α and IFN‐γ, than young Treg cells; however, aged Treg cells in the decidua produced higher levels of proinflammatory cytokines, such as TNF‐α and IFN‐γ, and lower levels of anti‐inflammatory cytokines, such as TGF‐β and IL‐10, than young Treg cells. Components of SASP can induce senescence of nearby non‐senescent cells in a paracrine manner, thereby increasing the overall number of senescent cells.^[^ ^28^ ^]^ In addition, abnormal mitochondria produce excessive ROS, which further activates the p38 MAPK pathway and enhances SASP secretion.^[^ ^29^ ^]^ CD4^+^ T cell senescence can induce chronic systemic inflammation and aggravate the systemic senescence phenotype,^[^ ^30^ ^]^ indicating that SASP may be both a result and cause of aging.

In this study, we also found that the proportion of PD‐1^+^ Treg cells in the decidua and peripheral blood of the Aged group in both women and mouse models was significantly increased when compared with that in the Young group, and the proportion of human PD‐1^+^ Treg cells was positively correlated with the proportion of human CD28^−^ Treg cells in the decidua. The confocal results also confirmed this viewpoint, as the colocalization of the aging markers p16 and PD‐1 expressed in Treg cells of the Aged group were significantly increased compared to that of the Young group of mice. Therefore, we speculate that PD‐1 may serve as a new surface marker of senescence in aged Treg cells, which may be more convenient to detect than the intranuclear markers p16 and p21.

Consistent with our results, a recent study using Jurkat T cells revealed that PD‐1 activation directly led to the dephosphorylation of the co‐stimulatory molecule CD28 and inhibited T cell function, indicating that PD‐1 increases the number of senescent CD28^−^ T cells.^[^ ^31^ ^]^ Additionally, single‐cell sequencing analysis of Treg cells from the lymphoid and adipose tissues of 3‐, 18‐, and 24‐month‐old mice revealed that the proportion of Treg cell subsets changed with increasing age. Of the six Treg cell subpopulations from C1 to C6, C3 and C5 may be young or immature Treg subpopulations, C2 and C4 may be in the transitional stage of aging, and C1 and C6 may be senescent subpopulations. A significant increase in PD‐1 expression is detectable only in the C4 Treg cell subpopulation. The mice selected for this study corresponded to middle‐aged humans (38–47 years old), which is similar to the characterization of the C4 subpopulation in the transitional stage of aging.^[^ ^32^ ^]^

MAPK signaling plays an important role in T cell senescence, and aged T cells display increased basal activation of MAPK signaling^[^ ^33^, ^34^ ^]^ Among the MAPK signaling pathways, the p38 MAPK pathway is a central signaling pathway that involves activating cell cycle regulatory molecules, such as p53, p21, and p16, which can inhibit cell cycle progression and thus slow or completely arrest DNA replication.^[^ ^29^ ^]^ In this study, we found that the p38 MAPK and inflammation‐related pathways were significantly enriched in aged PD‐1^+^ Treg cells, which might underlie the molecular mechanism of Treg cell aging. Compared to quiescent cells, growth‐promoting pathways, including mTOR and MAPK, have higher activity in senescent cells, and the role of the MAPK pathway in senescent cells is to accelerate aging rather than proliferation.^[^ ^35^ ^]^ Moreover, excessive activation of the p38 MAPK pathway leads to T cell aging and immune deficiency in humans.^[^ ^36^ ^]^ These results suggest that in advanced‐age pregnancies, high PD‐1 expression may cause Treg cell senescence through MAPK overactivation. Aging Treg cells have reduced inhibitory function and produce more proinflammatory cytokines, leading to impaired placental development and involvement in adverse pregnancies.

Adoptive transfer of immune cells has shown great potential in cancer treatment.^[^ ^37^ ^]^ Recently, studies have shown that adoptive transfer of Treg cells improves embryo resorption rates in abortion‐prone mouse models.^[^ ^38^, ^39^, ^40^ ^]^ Lewis et al.^[^ ^40^ ^]^ found that when splenic Treg cells from normal pregnant mice at E14 were transferred to CBA mice (an abortion‐prone mouse strain) at E2, the resorption rate significantly decreased. Similarly, our previous studies found that adoptive transfer of Blimp‐1^+^ or IL‐27‐treated Treg cells to an LPS‐induced abortion‐prone mouse model effectively reduced the embryo resorption rate and improved placental vascular development by promoting the accumulation of Treg cells in the decidua and the expression of negative signaling molecules such, as Tim‐3 and PD‐1, in Treg cells.^[^ ^38^ ^]^ Hence, the positive effects of the adoptive transfer of Treg cells on reducing embryo absorption in mice provide insights for improving adverse pregnancy outcomes in older pregnant mice.

Treg cells are the first and most severely senescent subpopulation of T cells, and senescent Treg cells can also induce the aging of other T cells. Tconv cells co‐cultured with senescent Treg cells express high levels of the cell aging marker β‐galactosidase and low levels of the co‐stimulatory molecules CD27 and CD28, and their cell cycle stagnates in the G0/G1 phase.^[^ ^34^, ^41^ ^]^ Therefore, adoptive transfer of Treg cells from young mice to older pregnant mice is expected to improve adverse pregnancy outcomes in older pregnant mice. The results of this study showed that primary Treg cells isolated from the spleens of young mice reduced the proportion of abnormal fetal mice at E11.5 in the aged recipient mice.

We labeled transferred Treg cells with carboxyfluorescein succinimidyl ester (CSFE) and found that the transferred young Treg cells were distributed to various organs and tissues in older mice. FCM was used to detect cytokine levels secreted by Treg cells in the spleen and placenta of older pregnant mice at E11.5. The secretion of the proinflammatory cytokine IFN‐γ by Treg cells in the spleen decreased, which may explain the improvement in pregnancy outcomes. In addition, this study found that blocking PD‐1 signaling reduced the expression of the aging marker p16 in older Treg cells and enhanced its ability to inhibit Tconv cell proliferation. To increase the clinical feasibility, we also explored the addition of autologous cell culture followed by reinfusion. Surprisingly, when aged Treg cells were pretreated with α‐PD‐1 mAbs and transferred to aged pregnant mice, the proportion of abnormal fetal mice markedly decreased. This finding provides new insights into future strategies by using autologous Treg cells pretreated with α‐PD‐1 mAbs to rescue adverse outcomes in AMA pregnancies.

In conclusion, this study discovered that Treg cells exhibited senescent phenotypes in reproductive‐aged women and mice and that PD‐1 may be correlated with Treg cell aging, which has not been previously reported. Transcriptome sequencing showed significant enrichment of MAPK and inflammation‐related (TNF‐α) pathways in the PD‐1^+^ Treg cells of aged mice. Moreover, the adoptive transfer of young Treg cells and aged Treg cells pretreated with α‐PD‐1 mAbs significantly reduced the proportion of abnormal fetal mice in the reproductive‐aged mouse model. Collectively, these findings shed new light on the potential mechanisms underlying immunosenescence and uncover potential therapeutic approaches to mitigate adverse pregnancy outcomes in older women. However, whether aging Treg cells in AMA pregnancy affect other immune cells and trophoblast cells at the maternal‐fetal interface requires in‐depth investigation.

## 4 Experimental Section

### Ethics Statement

Human study was approved by the Clinical Trial Ethics Committee of Huazhong University of Science and Technology (Wuhan, China; CTEC number: S154[2021]). All participants were recruited from the Department of Obstetrics and Gynecology of the Maternal and Child Health Hospital of Hubei Province, Wuhan, China. Mice study was approved by the Institutional Animal Care and Use Committee of Huazhong University of Science and Technology (Wuhan, China; IACUC number: 4051[2022]).

### Human Sampling

First trimester decidual tissue (6–9 weeks of gestation) was obtained from women with voluntary pregnancy terminations (termination for non‐medical reasons) who were categorized into two groups: Young (20–34 years of age) and Aged (35–45 years of age). In all patients, the menstrual cycle was regular and ranged from 27 to 30 days. None of the patients received hormonal treatment for at least 3 months before the procedure or had anatomic abnormalities. Peripheral blood samples were obtained from women with clinically normal pregnancies during the first trimester. The blood samples were placed into Falcon tubes (50 mL) containing PBS with sodium citrate (15 mM) and 10% antibiotic–antimycotic solution and transferred to the lab at 4 °C within 2 h. The blood samples were centrifuged at 800 ×g for 5 min, the supernatant was removed, and the pellets were placed in Petri dishes in DMEM/F12 containing 10% antibiotic‐antimycotic solution. The decidual tissues were diced with a scalpel and then digested with collagenase I (0.5 mg mL^−1^) and DNase (0.1 mg mL^−1^) for 1 h at 37 °C in a humidified incubator under 5% CO_2_. After enzymatic digestion, the tissues were centrifuged at 400 ×g for 5 min. The suspensions were loaded onto a Ficoll density gradient to purify the lymphocytes. Subsequently, the decidual immune cells were collected.

### Mice

All animal procedures were performed in accordance with the guidelines of the Institutional Animal Care and Use Committee of Tongji Medical College, Huazhong University of Science and Technology, Wuhan, China.

Heterozygous young Pdcd1^+/−^ mice (C57BL/6J background, provided by Cyagen Biosciences) mice were mated with each other, and toe or tail tissues were collected from offspring at 21 days for genetic analysis. PCR was performed using tissue DNA with the primers detailed in Table S1 (Supporting Information). PCR products were analyzed using 1.5% agarose gel electrophoresis, including blank, WT (single band at 532 bp), PD‐1^+/−^ (bands at 532 and 453 bp), and PD‐1^−/−^ (single band at 453 bp) groups.

Aged female C57BL/6 mice (40–48 weeks) were mated with BALB/c males (8–16 weeks) (both purchased from the Animal Center of Tongji Medical College), and the presence of a vaginal plug indicated a gestational day of 0.5 (E0.5). C57BL/6 dams were randomly divided into three groups: Y‐A group, tail vein injection of 2×10^5^ Treg cells from the spleens of mice in the Young group in RPMI 1640 (200 µL) on E6.5 and E7.5; A‐A group, tail vein injection of 2×10^5^ Treg cells from the spleens of mice in the Aged group in RPMI 1640 (200 µL) at E6.5 and E7.5; and AP‐A group, tail vein injection of 2×10^5^ Treg cells from the spleens of mice in the Aged group that were pretreated with anti‐PD‐1 mAbs in RPMI 1640 (200 µL) at E6.5 and E7.5. MACS kits (Miltenyi Biotec) were used to select CD4^+^CD25^+^ T cells from the spleen cells of young and aged female C57BL/6 mice for adoptive transfer. The embryo abortion rate, cytokine secretion in the spleen and uterus, and placental development were evaluated at E11.5.

### Isolation of Mouse Spleen Primary Treg Cells

MACS kits (Miltenyi Biotec) were used to select CD4^+^CD25^+^ T cells from the spleen cells of nonpregnant female C57BL/6 mice. Both sets of cells were detected using FCM and stained with fluorescein isothiocyanate (FITC)‐anti‐CD4 and phycoerythrin (PE)‐anti‐CD25 antibodies. Freshly isolated Treg cells were treated with anti‐PD‐1 mAb (10 µg mL^−1^).

### Coculture of Aged T‐Conv Cells with Treg cells

Primary Treg cells (CD4^+^CD25^+^) and aged T‐conv cells (CD4^+^CD25^–^) were isolated from mouse spleen by MACS. CFSE‐labeled aged splenic T‐conv cells (1 × 10^5^) were cocultured with an equal number (1 × 10⁵) of Treg cells from different experimental groups in 24‐well plates for 5 days. The proliferation of aged T‐conv cells was assessed by FCM.

### RNA Sequencing Data Analysis

Freshly sorted Young and Aged CD4^+^CD25^+^PD‐1^+^ (n = 3) or CD4^+^CD25^+^PD‐1^−^ (n = 3) mouse spleen Treg cells (>10^5^ cells) were lysed using TRIzol reagent (Life Technologies, USA). Transcriptome sequencing was conducted by Beijing Nuohuzhiyuan Company, with effective library concentrations exceeding 1.5 nM. Sequencing was performed using an Illumina NovaSeq 6000, with a data volume per sample exceeding 5 Gb. The Young and Aged groups underwent differential expression analyses, and the resulting genes were subjected to GO and KEGG enrichment analyses.

### FCM

FCM was performed using the NovoCyte system (Agilent, USA), and the obtained data were analyzed using NovoCyte version 2.0. Intracellular ROS levels were assessed using DCFH‐DA dye (Beyotime, China). For measurements of human and mouse intranuclear antigens p53, p21, and 16, single‐cell suspensions were pretreated with the True‐Nuclear Transcription Buffer Set (BioLegend, USA). For measurements of the human and mouse intracellular antigens IL‐6, TNF‐α, IFN‐γ, IL‐10, and TGF‐β, single‐cell suspensions were pretreated with the Cell Activation Cocktail (BioLegend, USA) and Fixation/Permeabilization kit (BD Biosciences, USA). Naïve Treg cells were labeled as CD4^+^CD25^+^CD127^−^CD45RA^+^; memory Treg cells were labeled as CD4^+^CD25^+^CD127^−^CD45RA^−^, and activated memory Treg cells were labeled as CD4^+^CD25^+^CD127^−^HLA‐DR^+^CD45RA^−^. All fluorescent antibodies used were listed in Table S2 (Supporting Information).

### Immunofluorescence

Treg cells from the spleens of mice in the Young and Aged groups were fixed in 4% formaldehyde for 15 min, permeabilized with 0.1% Triton X‐100 for 10 min, and blocked with 1% bovine serum albumin for 1 h. Cells were incubated with primary anti‐PD‐1 antibodies (Abcam, #ab52587) and a P16‐INK4A polyclonal antibody (Proteintech, Cat No. 10883‐1‐AP) overnight at 4 °C, followed by the incubation with secondary antibodies, CoraLite 488‐conjungated goat anti‐rabbit IgG (Proteintech, China) and CoraLite 594‐conjungated goat anti‐mouse IgG (Proteintech, China), for 1 h at room temperature. The slides were counterstained with DAPI (1 µg/ mL), mounted using 2% propyl gallate, and analyzed using an Apotome 3 microscope (ZEISS, Germany).

### RNA Extraction, Reverse Transcription, and Real‐Time PCR

For RNA extraction, the placental tissue was lysed using TRIzol reagent (Life Technologies, USA). The purity and concentration of the RNA samples were assessed spectrophotometrically using a NanoDrop 2000 (Thermo Scientific). Reverse transcription was performed using T30 (LongGene, China). Gene expression levels were assessed using the Taq Pro Universal SYBR qPCR Master Mix (Q712; Vazyme, China) on a Quantgene q225‐0307 instrument (Kubo Technology, China). The primer sequences are listed in Table S1 (Supporting Information). β‐actin was used as the reference gene.

### Statistical Analysis

All statistical analyses were performed using GraphPad Prism 9.0. All quantitative data are presented as mean ± SEM. *p*‐values < 0.05 were considered statistically significant. A t‐test was used to determine the significance of differences between two groups. A one‐way analysis of variance (ANOVA) was used to compare multiple groups. Pearson's correlation analysis was used to analyze the linear relationship between two variables.

## Conflict of Interest

The authors declare no conflict of interest.

## Author Contributions

G.‐S. G., Y.‐J. Z., X.‐H. H., contributed equally to this work. G.G. and Y.Z. designed the experiments and analyzed the data. G.G. and X.H. wrote the manuscript. X.L. provided *Pdcd1^−/−^* mice and samples. A.L. contributed to the concept, design, supervision, results’ interpretation, text editing and final approval. All authors discussed the results and implications and commented on the manuscript at all stages.

## Supporting information



Supporting Information

## Data Availability

The data that support the findings of this study are available from the corresponding author upon reasonable request.
