# Supplementary material for: PD‐1‐Enhanced Treg Cell Senescence in Advanced Maternal Age
Source: Adv Sci (Weinh). 2024 Dec 23;12(6):2411613. doi: 10.1002/advs.202411613 (PMC11809324; doi:10.1002/advs.202411613)
Supplement: Supplementary file 1 — Supporting Information [file ADVS-12-2411613-s001.docx]

**Supplementary table 1. Real-time PCR gene primer sequences.**

| Gene target | Forward primer (5’ – 3’) | Reverse primer(3’ – 5’) |
| --- | --- | --- |
| *Pdcd1*-KO | GTTGCCAAATGACTACATCGGC | ATCTCAACCCTTCCCTATGCTG |
| *Pdcd1*-WT | GTTGCCAAATGACTACATCGGC | CCCTGAGTAGCAGAAGGCATAG |
| m-*P16* | AATCTCCGCGAGGAAAGC | GTCTGCAGCGGACTCCAT |
| m-*P21* | GTCAGGCTGGTCTGCCTCCG | CGGTCCCGTGGACAGTGAGCAG |
| m-*P53* | CATCACCTCACTGCATGGAC | TGAGGGGAGGAGAGTACGTG |
| m-*IL-6* | CAAAGCCAGAGTCCTTCAGA | GATGGTCTTGGTCCTTAGCC |
| m-*TNFα* | TCTTCTCATTCCTGCTTGTGG | GGTCTGGGCCATAGAACTGA |
| m-*IL-10* | AGGCGCTGTCATCGATTTCTC | GACACCTTGGTCTTGGAGCTTAT |
| m-*IFNγ* | TTGCCAAGTTTGAGGTCAACAAC | CGAATCAGCAGCGACTTCTT |
| m-*IL-1β* | GCAACTGTTCCTGAACTCAACT | ATCTTTTGGGGTCCGTCAACT |
| m-*TGFβ1* | GCAACAATTCCTGGCGTTACC | CAGTGAGCGCTGAATCGAAAG |
| m-*actin* | GGC TGT ATT CCC CTC CAT CG | CCAGTTGGTAACAATGCCATGT |

**Supplementary table 2. Flowcytometry antibody information.**

| Label- Reactivity - Name | Cat. No. | Brand - Country |
| --- | --- | --- |
| FITC anti-human CD4 Antibody | 300506 | Biolegend, USA |
| APC anti-human CD4 Antibody | 317415 | Invitrogen, USA |
| PE anti-human CD25 Antibody | 302606 | Biolegend, USA |
| APC anti-human CD127 Antibody | 351316 | Biolegend, USA |
| APC/cy7 anti-human CD127 Antibody | 351350 | Biolegend, USA |
| PE/cy7 anti-human PD-1 | 3229918 | Biolegend, USA |
| PE/cy7 anti-human CD28 | 302926 | Biolegend, USA |
| PERCP anti-human HLA-DR | 307628 | Biolegend, USA |
| APC anti-human CD45RA | 304128 | Biolegend, USA |
| APC anti-human p21 AF647 Antibody | SC-6246AF647 | Santa, USA |
| APC anti-human p16 AF647 Antibody | SC1661AF647 | Santa, USA |
| APC anti-human p53 AF647 Antibody | 2533S | Cell Signalling Technology, USA |
| PE anti-mouse PD-1 Antibody | 135206 | Biolegend, USA |
| PE anti-mouse CD26 Antibody | 102008 | Biolegend, USA |
| PERCP anti-mouse CD44 Antibody | 103032 | Biolegend, USA |
| APC anti-mouse CD62L Antibody | 104411 | Biolegend, USA |
| FITC anti-mouse CD4 Antibody | 11-0041-82 | Invitrogen, USA |
| Anti-human TNF-α-APC | 502912 | Biolegend, USA |
| Anti-human IL-10-APC | 506807 | Biolegend, USA |
| Anti-human IFN-γ-APC | 502512 | Biolegend, USA |
| Anti-human TGF-β1-APC | 300006 | Biolegend, USA |
| Anti-mouse PD-1 | ab234444 | Abcam, USA |
| Anti-mouse CDKN2A | 10883-1-AP | Proteintech, China |
